# Computational design and molecular dynamics simulations suggest the mode of substrate binding in ceramide synthases

**DOI:** 10.1038/s41467-023-38047-x

**Published:** 2023-04-22

**Authors:** Iris D. Zelnik, Beatriz Mestre, Jonathan J. Weinstein, Tamir Dingjan, Stav Izrailov, Shifra Ben-Dor, Sarel J. Fleishman, Anthony H. Futerman

**Affiliations:** 1 Department of Biomolecular Sciences, Weizmann Institute of Science, Rehovot, 76100 Israel; 2 Life Sciences Core Facilities, Weizmann Institute of Science, Rehovot, 76100 Israel

**Keywords:** Molecular modelling, Computational models, Enzyme mechanisms, Computational biophysics

## Abstract

Until now, membrane-protein stabilization has relied on iterations of mutations and screening. We now validate a one-step algorithm, mPROSS, for stabilizing membrane proteins directly from an AlphaFold2 model structure. Applied to the lipid-generating enzyme, ceramide synthase, 37 designed mutations lead to a more stable form of human CerS2. Together with molecular dynamics simulations, we propose a pathway by which substrates might be delivered to the ceramide synthases.

## Abstract (web summary)

Membrane proteins are involved in many critical cellular pathways. Here, authors use a combination of structural predictions, an algorithm for stabilizing membrane proteins, and molecular dynamics to reveal a putative mechanism for the action of ceramide synthases.

## Introduction

A quarter of the human genome encodes integral membrane proteins^1^, with a significant number of these spanning the membrane multiple times. It has proved difficult to resolve the three-dimensional structure of such proteins by either X-ray crystallography or by cryo-electron microscopy^2^, such that <2% of proteins in the protein data bank (PDB) are membrane proteins^3^. The recent publication of the AlphaFold2^4^ algorithm has dramatically increased the number of reliable protein structural models^5^, including multi-spanning membrane proteins.

**Fig. 1:**
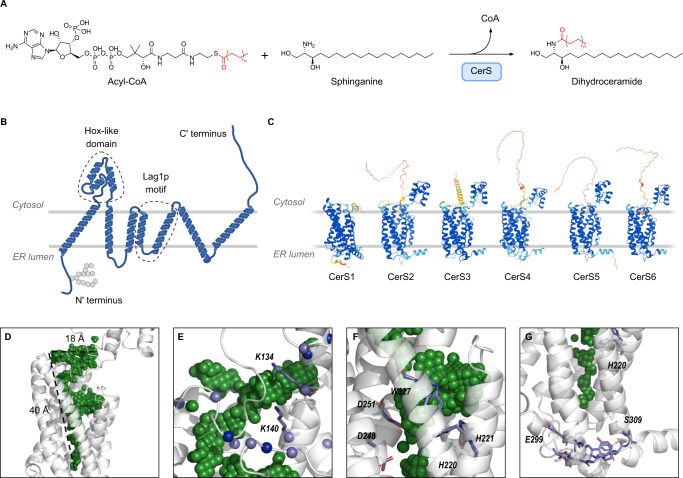
AlphaFold2 predictions of the structure of the six mammalian CerS. **A** Reaction scheme for CerS activity in the ceramide de novo synthesis pathway. **B** Putative topology of the CerS based on AlphaFold2 predictions. The Hox-like, Lag1p domain, and the N-terminal glycosylation site are indicated. **C** AlphaFold2 models of human CerS1-6. Note that CerS1 lacks a Hox-like domain. Models are colored by structure prediction confidence as estimated by predicted Local Distance Difference Test (pLDDT) (dark blue, pLDDT >90; light blue, pLDDT of 90–70; yellow, pLDDT of 70–50; orange, <50. **D**–**G** CerS5 AlphaFold2 predicted structures showing cavities in the TMD. Protein in gray, cavity volume in green spheres. **D** The funnel-shaped cavity observed in the CerS5 structure shows a wide opening at the cytoplasmic end of the TMD which narrows towards the ER lumenal end of the TMD. **E** Positively charged amino acids surround the cytoplasmic end of the TMD in CerS5. Lysine and arginine Cɑ atoms shown as spheres (light blue, lysine; dark blue, arginine). **F** Putative CerS5 catalytic site showing key residues in stick representation. **G** The CerS5 specificity loop near the cavity in the TMD. The 11-residue specificity sequence is shown in stick representation with the residues at the beginning (E299) and at the end (S309) indicated.

Many proteins involved in lipid metabolism and transport are multi-spanning membrane proteins located in the endoplasmic reticulum (ER). In mammals, ceramide, a critical structural and signaling lipid, is synthesized by one of six homologous membrane-bound enzymes, namely the ceramide synthases (CerS)^6^. The key feature distinguishing CerS isoforms is their specific use of fatty acyl-CoAs of different acyl chain lengths (Fig. 1A). Thus, CerS2 uses C22-C24-acyl-CoAs whereas CerS5 uses C16-CoA to *N*-acylate sphingoid long-chain bases (LCBs) such as sphinganine (d18:0) and sphingosine (d18:1).

Here, we study the AlphaFold2 CerS structure using molecular dynamics to demonstrate this structural model is a reliable framework for mutational analysis and atomistic design calculations.

## Results and discussion

### Structure predictions of CerS

Although we have recently been able to solubilize CerS5 in a partially active form, we were unable to obtain enough material to determine the three-dimensional structure by cryo-electron microscopy at a high enough resolution. Thus, we persued a computational approach by comparing the predicted structures of CerS5 using RaptorX^7^, trRosetta^8^, RoseTTAfold^9^, and AlphaFold2^4,10^. All four programs predicted a structure with a bundle of seven alpha-helices of ~30–50 Å length, with the N-terminus on one side of the membrane and the C-terminus and the Hox-like domain^11^ on the other side (Fig. 1B), consistent with earlier predictions^12^. The AlphaFold2 structures of the six human CerS enzymes are very similar (Fig. 1C and Supplementary Table 1), which is expected in light of their high sequence similarity (Supplementary Table 2). The structure of the Hox-like domain is similar to the experimentally-resolved structure (RMSDs of 0.9 Å and 0.6 Å to murine CerS5 [PDB: 2CQX] and CerS6 [PDB: 1X2M]).

Based on the AlphaFold2 CerS structure, we propose a binding mode for acyl-CoA in a funnel-like crevice of ~40 Å length (Fig. 1D) within the transmembrane domain (TMD). The funnel entrance at the cytoplasmic face contains numerous conserved positively charged residues^11^ (Fig. 1E) which may interact with the three negatively charged phosphates in the CoA moiety, similar to the CoA-binding interactions in human diacylglycerol acyltransferase^13^. Among residues at the funnel entrance are K134 and K140 (numbering corresponds to human CerS5), in which mutations to alanine reduce CerS activity by >50%^11^, while mutations to arginine maintain activity, illustrating the need for a positive charge in this site. Several conserved residues between all CerS^14^ (H220, H221, D248, D251, and W327 in CerS5), which are critical for catalytic activity (ref. ^15^ and Supplementary Fig. 1), are found at the mid-point of the funnel (Fig. 1F). Toward the luminal face of the enzyme, the funnel narrows and contains mainly hydrophobic residues which match the size and hydrophobicity of the acyl chain of acyl-CoA. Finally, the 11 residues between the sixth and seventh TM helices, which alter CerS acyl-CoA specificity^16^, are proximal to the narrow end of the funnel (Fig. 1G).

### Computer-aided design of a thermostable CerS2

In order to verify the atomic accuracy of the AlphaFold2 models, we designed CerS variants using mPROSS (membrane-Protein Repair One Stop Shop^17^), a membrane-protein stability method that encodes a membrane-embedded energy function and uses phylogenetic analysis of homologs to select stabilizing mutations that are more commonly observed in a multiple-sequence alignment of homologs^18–20^ (the mPROSS server [http://mPROSS.weizmann.ac.il] provides automated access to this workflow). To account for modeling uncertainty, mutations were not allowed in low-confidence regions, identified according to the AlphaFold2 plDDT scores and neighboring positions. Four designs were generated for CerS2 and three for CerS5 (Supplementary Fig. 2). For CerS2, design 5 (d5) contained 7 mutations, whereas d18 contained 37 (~10% of the sequence). Even though the active site was not explicitly restricted, mPROSS avoided mutating any active site positions, as these are implicitly recognized by the phylogenetic analysis. Rather, mPROSS mutated positions across the rest of the protein, including both cytosolic and transmembrane domains. Such mutations increase hydrophobicity of the membrane domain, improve interhelical packing, and enhance solubility in the cytoplasmic domain (Supplementary Fig. 3).

**Fig. 2:**
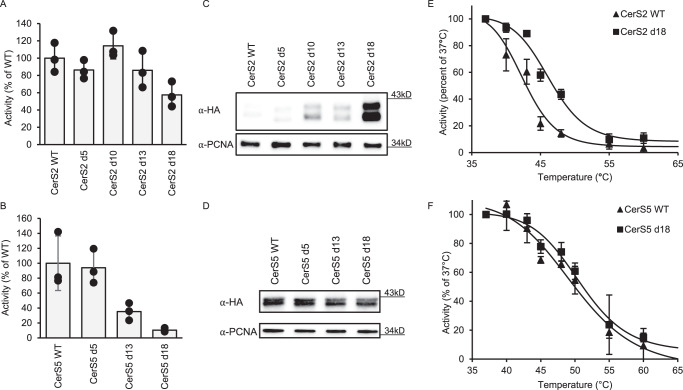
Gain of function mutations in CerS2 introduced by mPROSS. Homogenates were prepared from **A** HEK^CerS2−/−^ cells overexpressing the indicated CerS2 designs and **B** WT HEK cells overexpressing the indicated CerS5 designs. Results are shown as a percent of the activity in cells overexpressing the WT enzyme; means ± S.D. *n* = 3. Expression levels of CerS2 **C** or CerS5 **D** designs in WT HEK cells were ascertained by western blotting using an anti-HA antibody and anti-PCNA as a loading control. Molecular weight markers are indicated. Results are of a single experiment, repeated three times with similar results. Thermal stability of WT and d18 CerS2 (**E**) and CerS5 (**F**) were determined after incubation of homogenates at increasing temperature for 20 min, followed 30 min later by assay of CerS activity at 37 °C. Results are shown as percent of activity at 37 °C; means ± S.D. *n* = 3. Source data are provided as a Source Data file.

Remarkably, despite the fact that site-directed mutagenesis often leads to loss of CerS activity and/or expression^6^, CerS2 d18 maintained ~60% of wild-type (WT) activity, while activity was similar to WT CerS2 for the other designs (Fig. 2A). Moreover, expression of d18 CerS2 increased 12.0 ± 4.95-fold compared to the WT (Fig. 2C). In contrast, CerS5 lost activity upon increasing the number of mutations (Fig. 2B), with no effect on protein expression (Fig. 2D). It should be noted that the basal activity of WT CerS5 is much higher than that of CerS2 [5723 ± 2092 pmol C16-ceramide/min/mg protein (CerS5) compared to 56.3 ± 9.5 pmol C22-ceramide/min/mg protein (CerS2)], perhaps suggesting that this enzyme is better optimized and therefore less amenable to the mutations introduced by mPROSS; moreover, the specific activity of d18 CerS5 (570 ± 122 pmol C16-ceramide/min/mg protein) is still considerably higher than that of WT CerS2. The acyl-CoA specificity of mPROSS CerS2 designs was unchanged (Supplementary Fig. 4). However, the thermal stability of d18 CerS2 was ~4 °C higher than WT CerS2 (46.2 °C ± 0.11 versus 42.6 °C ± 0.02) (Fig. 2E), whereas the thermal stability of d18 CerS5 did not differ from that of WT CerS5 (50.3 °C ± 2.0 versus 51.6 °C ± 0.2) (Fig. 2F). These results show that the AlphaFold2 structure of CerS2 is a reliable framework for mutational analysis and even for atomistic design calculations.

### Molecular dynamics simulations suggest substrate access routes

**Fig. 3:**
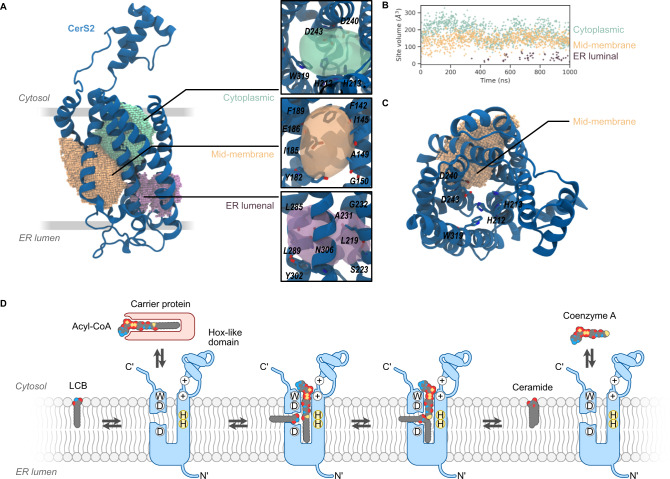
MD simulations of CerS2 reveal putative binding pockets and suggest a working model for *N*-acyltransferase activity. **A** The three pockets in the cytoplasmic (*green*), mid-membrane (*orange*), and ER lumen (*purple*) are shown as spheres based on SiteMap pocket predictions. The inset (*right*) depicts the position of the three pockets as translucent isosurfaces with selected residues labeled and shown as sticks with per-atom coloring. **B** Volume of pockets plotted *versus* time of simulation. Vertical axis shows cavity volume (Å^3^). Source data are provided as a Source Data file. **C** Vertical view of CerS2 with the mid-membrane pocket shown as spheres. Highly conserved residues lining the predicted active site are labeled. **D** Working model for CerS *N*-acyltransferase activity. The CerS active site accommodates an acyl-CoA, which is delivered from the cytoplasmic side of the ER membrane via acyl-CoA carrier proteins. Sphingoid bases access the active site via a side channel that accommodates the sphingoid motif but leaves the acyl tail free in the hydrophobic region of the bilayer. Acyl chain transfer is proposed to occur once both molecules are present in the active site, coordinated by the conserved double histidine motif. After ceramide synthesis, the product is released to the bilayer, while the free CoA is released to the cytoplasm. CerS in *blue* with N- and C-termini, and the Hox-like domain indicated. The position of the conserved tryptophan, aspartic acids, and histidine residues are indicated using one-letter codes, while positively charged residues are indicated with circled plus signs. The LCB, acyl-CoA, and ceramide are indicated with space-filling cartoons colored by atom type.

We next analyzed the volume of the funnel-like crevice of CerS2 by molecular dynamics (MD) simulations in an ER-like membrane (see Supplementary Table 3 for lipid composition). MD simulations allow the detection of the motion of the protein backbone and amino acid side chains, providing a more accurate view of hydrophobic pockets than a single static predicted structure as produced by AlphaFold2. During simulations (1 µs), the TMDs retained their tertiary structure (Supplementary Fig. 5). Three pockets were detected (Fig. 3A), located at the cytoplasmic and ER lumenal ends of the TMDs as well as in a mid-membrane region between helices 2 and 3 (Fig. 3A). The volumes of two of the pockets are sufficiently large to accommodate acyl-CoA (Fig. 3B; average volume for the cytoplasmic-facing pocket is 186 Å^3^; average volume for the mid-membrane pocket is 141 Å^3^). The third pocket, located at the ER lumenal end of the TMD is smaller (average volume 38 Å^3^). The cytoplasmic pocket is close to conserved W319 and to H212 and H223, which may be involved in catalytic activity^15^ (see also Supplementary Fig. 1). The mid-membrane pocket, located between helices 2 and 3, forms a channel between the surrounding bilayer and these conserved residues (Fig. 3C) whereas the smaller ER lumenal pocket is nestled at the base of helices 5, 6 and 7 (Fig. 3A).

The two larger pockets at the cytoplasmic and mid-membrane areas suggest two routes of access to the putative catalytic site, and we propose that they provide an entry route for each of the two substrates. The sphingoid LCB is generated in the ER membrane^21^, whereas acyl-CoA is generated in the cytoplasm and supplied to the CerS via acyl carrier proteins^22^; we now suggest that the former gains access to the active site via the mid-membrane pocket (Fig. 3D), whereas acyl-CoA is delivered to the cytoplasmic pocket where it interacts with the positively charged amino acids located near the junction of the Hox-like domain and the TMD. This is consistent with studies showing that CerS are highly specific with respect to the use of acyl-CoAs but less specific about the type of LCB; for instance, NBD-sphinganine can be used for catalysis but NBD-acyl-CoA cannot^23^. In the substrate-bound configuration, the acyl chain moiety of acyl-CoA occupies the ER lumenal pocket. This binding mode places the reactive regions of the two substrates (the acyl-CoA thioester and the sphingoid motif^24^ of the LCB) in immediate proximity with the proposed catalytically-active histidine residues. After the transfer of the acyl chain to the LCB, the newly generated ceramide would be released to the ER bilayer via the mid-membrane pocket while the CoA returns to the cytoplasm. This orthogonal arrangement of substrate entry routes has been observed in two recently solved acyltransferase structures^13,25^. In both cases, the acyl-CoA-binding pocket is oriented perpendicular to the membrane surface, while the pocket containing the second lipid substrate is parallel to the membrane surface.

## Methods

### Materials

NBD-Sphinganine (NBD-Sph) and fatty acyl-CoAs were from Avanti Polar Lipids (Alabaster, AL). Defatted BSA, a protease inhibitor mixture, and polyethyleneimine were from Sigma. An ECL detection system and a BCA reagent kit were from Cyanagen (Bologna, Italy). Silica gel 60 TLC plates were from Merck (Billerica, MA). All solvents were of analytical grade and were purchased from Bio-Lab (Jerusalem, Israel).

### Bioinformatics

Alignment of human CerS sequences was performed with Muscle 3.81.31^26^. Similarity and identity percentages were calculated with MacVector version 18.0. Alignment of mPROSS designs and WT CerS2 and CerS5 were performed with CLUSTALW 2.1^27^.

### CerS constructs

CerS5 W327A was subcloned from CerS5 in a pcDNA3.1 vector carrying a C-terminal HA tag, using restriction-free cloning^28^ with the following primers: CAATTAGGTAGGACGCGATGACATGCAGAAG and CTTCTGCATGTCATCGCGTCCTACCTAATTG. The sequence was confirmed prior to use. All mPROSS CerS2 and CerS5 constructs (including WT) were synthesized with a C-terminal HA tag and cloned in a pcDNA3.1+ vector by GenScript after modifying and optimizing the coding sequence for use with a mammalian expression system (http://www.genscript.com/gene_synthesis.html).

### mPROSS algorithm

The ColabFold server was used to model both CerS2 and CerS5, and the best ranking AMBER relaxed models were selected^4,29^. The mPROSS algorithm was applied to both models, while restricting all positions with plDDT confidence scores <0.90^30^. Additionally, for every stretch of low-confidence positions, two adjacent primary sequence positions were also restricted. Details of the mPROSS algorithm and server have been described^17^. Briefly, mPROSS applies a phylogenetic analysis to generate a PSSM (Position Specific Score Matrix^31^) based on a multiple-sequence alignment of homologs. At each position, mutations that exhibit a PSSM score <0 are eliminated from further consideration. Furthermore, Rosetta atomistic design calculations model each of the remaining mutations, and ones that are not stabilizing are also eliminated. 18 energy thresholds are used to generate 18 sequence spaces. Finally, Rosetta combinatorial design calculations are applied to search the lowest-scoring combination of mutations in each sequence space, generating designs that span a wide range from very conservative (a few mutations) to very promiscuous (many mutations). All Rosetta calculations use the ref2015_memb energy function which uses the ref2015 all-atom soluble energy function in the cytoplasmic domain and a membrane energy function with membrane-depth and burial-dependent lipophilicity terms^32^. The energy function transitions gradually from cytoplasmic to membrane domains. Designs are then manually chosen for experimental characterization.

### Cell culture and transfection

WT HEK293T (HEK, ATCC, CRL-3216) or CRISPR CerS2 KO HEK293T cells (HEK^CerS2−/−^, see ref. ^16^) were cultured in Dulbecco’s modified Eagle’s medium supplemented with 10% fetal calf serum, 100 IU/ml penicillin, 100 µg/ml streptomycin and 110 μg/ml sodium pyruvate. Transfections were performed with the polyethyleneimine reagent using 4 μg of plasmid per 10 cm culture dish. 48 h after transfection, cells were removed from culture dishes and washed twice with PBS. Cell homogenates were prepared in 20 mM Hepes-KOH, pH 7.2, 25 mM KCl, 250 mM sucrose, and 2 mM MgCl_2_ containing a protease inhibitor cocktail. Protein was determined using the BCA reagent.

### Western blotting

Proteins were separated by SDS-PAGE and transferred to nitrocellulose membranes by Trans Blot Turbo (Bio-Rad). HA-tagged constructs were identified using a rabbit anti-HA antibody (Sigma, H6908, 1:10,000) and goat anti-rabbit horseradish peroxidase (Jackson ImmunoResearch, 115-035-003, 1:5000) as the secondary antibody. Equal loading was confirmed using a mouse anti-tubulin (Sigma, T9026, 1:10,000) or mouse anti-PCNA (Santa Cruz, SC-56, 1:500) antibody and goat anti-mouse horseradish peroxidase (Jackson ImmunoResearch, 111-035-003, 1:5000) as the secondary antibody. Detection was performed using the ECL detection system. Uncropped and unprocessed scans are provided in the Source Data file.

### Ceramide synthase assays and thermostability measurements

Cell homogenates were incubated with 15 µM NBD-sphinganine, 20 µM defatted BSA, and 50 µM fatty acyl-CoA in a 20 µl reaction volume at 37 °C. Activity was assayed using 40 µg protein and C22-CoA for 25 min for CerS2 and 2 µg of protein and C16-CoA for 5 min for CerS5. Reactions were terminated by addition of chloroform/methanol (1:2, v/v) and lipids extracted^33^. Lipids were dried under N_2_, resuspended in chloroform/methanol (9:1, v/v), and separated by thin layer chromatography using chloroform/methanol/2 M NH_4_OH (40:10:1, v/v/v) as the developing solvent. NBD-labeled lipids were visualized using an Amersham™ Typhoon™ Biomolecular Imager and quantified by ImageQuantTL (GE Healthcare, Chalfont St Giles, UK). For thermostability measurements, cell homogenates were incubated at various temperatures for 20 min followed by 30 min on ice. CerS activity was measured at 37 °C as above. All raw data are provided in the Source Data file.

### MD simulations

CerS2 coordinates were obtained from the AlphaFold2 database (UniProt ID: Q96G23). The lipid bilayer was constructed using CHARMM-GUI^34^ based on published lipidomics data^35^ (Supplementary Table 3). The simulation contained 396 lipid molecules, 206 potassium ions, 110 chloride ions, and 40,780 TIP3P water molecules in a rectilinear box (10 nm × 10 nm × 15 nm). Equilibration was performed in an NVT ensemble for 250 ps, and subsequently in the NPT ensemble for 500 ps using the Berendsen thermostat (310 K) and barostat (1 atm), and finally for 10,000 ps using the Nose-Hoover thermostat and Parrinello-Rahman barostat. Production simulation was performed for 1 μs. Simulation was performed using Gromacs 2020 (source code archive 10.5281/zenodo.3562495)^36^ using the CHARMM36 forcefield^37^ available through CHARMM-GUI. Binding pockets were detected using SiteMap from Schrödinger, Inc (Schrödinger Suite version 2021-4 build 135)^38^ using a pocket report size of 10 site points. Molecular graphics were produced using VMD 1.9.3^39^ and PyMOL 2.3.5^40^ Graphs were generated using open-source software (Python 3.8.5)^41^.

### Reporting summary

Further information on research design is available in the Nature Portfolio Reporting Summary linked to this article.

## Supplementary information

Supplementary Information

Reporting Summary

## Data Availability

Human CerS three-dimensional models are available at [https://alphafold.ebi.ac.uk/] under the following UniProt accession codes: CerS1, P27544; CerS2, Q96G23; CerS3, Q8IU89; CerS4, Q9HA82; CerS5, Q8N5B7; CerS6, Q6ZMG9. The accession codes for the PDB structures of the Hox-like domains are CerS5, PDB: 2CQX and CerS6, PDB: 1X2M. The molecular dynamics simulation trajectory files and SiteMap analysis files are publicly available at [https://github.com/tamir-dingjan/CerS2], 10.5281/zenodo.7608937. The source data underlying Fig. 2, and Supplementary Figs. 1 and 4 are provided as a Source Data file. Source data are provided with this paper.

## Source data

Source Data

## Code availability

The mPROSS algorithm is available online at: [https://mPROSS.weizmann.ac.il]. The Python code used to generate manuscript Fig. 3B and Supplementary Fig. 5 are available at https://github.com/tamir-dingjan/CerS2 [10.5281/zenodo.7608937].
