# Supplementary material for: Computational design and molecular dynamics simulations suggest the mode of substrate binding in ceramide synthases
Source: Nat Commun. 2023 Apr 22;14:2330. doi: 10.1038/s41467-023-38047-x (PMC10122649; doi:10.1038/s41467-023-38047-x)
Supplement: Supplementary file 1 — Supplementary Information [file 41467_2023_38047_MOESM1_ESM.pdf]

SUPPLEMENTARY INFORMATION

Computational design and molecular dynamics simulations suggest the mode of substrate binding in ceramide synthases

Iris D. Zelnik[1], Beatriz Mestre[1], Jonathan Weinstein[1], Tamir Dingjan[1], Stav Izrailov[1], Shifra Ben-Dor[2], Sarel J Fleishman[1] and Anthony H. Futerman[1 *]

From the [1]Department of Biomolecular Sciences and [2]Life Sciences Core Facilities, Weizmann Institute of Science, Rehovot 76100, Israel

[*] To whom correspondence should be addressed at tony.futerman@weizmann.ac.il

Contents

Supplementary Table 1. RMSDs for AlphaFold2 CerS models.
Supplementary Table 2. Percent sequence similarity and identity between human CerS proteins.
Supplementary Table 3. Composition of the ER-like membrane used in MD simulations.
Supplementary Figure 1. The highly conserved residue, Trp 327 is critical for CerS activity.
Supplementary Figure 2. Sequence alignment of WT and mPROSS designs.
Supplementary Figure 3. CerS stabilization by mPROSS.
Supplementary Figure 4. Acyl-CoA specificity of CerS2 d18 is similar to the WT.
Supplementary Figure 5. RMSD of the TMDs of CerS2 during production-stage MD simulation.
Supplementary References

Supplementary Table 1. RMSD values (Å) of AlphaFold2 CerS models for superimposition of the structures of the full-length protein (above the diagonal) and for the TMDs (below the diagonal).

	CerS1	CerS2	CerS3	CerS4	CerS5	CerS6	Full length-Protein RMSD
CerS1		2.02	1.35	0.86	2.18	1.72	CerS1
CerS2	1.06		0.53	0.56	0.67	0.64	CerS2
CerS3	0.93	0.37		0.74	0.66	0.80	CerS3
CerS4	0.75	0.39	0.41		0.56	0.59	CerS4
CerS5	0.95	0.47	0.50	0.46		0.43	CerS5
CerS6	0.88	0.51	0.52	0.51	0.30		CerS6
TMD RMSD	CerS1	CerS2	CerS3	CerS4	CerS5	CerS6	

Supplementary Table 2. Percent sequence similarity (below the diagonal) and identity (above the diagonal) between human CerS proteins.

	CerS1	CerS2	CerS3	CerS4	CerS5	CerS6	Identity
CerS1		21.8	20.4	24.4	23.2	24.6	CerS1
CerS2	35.6		52.1	47.6	40.1	40.9	CerS2
CerS3	35.8	70.7		42.3	39.0	38.5	CerS3
CerS4	37.0	66.3	61.0		40.4	40.5	CerS4
CerS5	35.4	57.9	57.0	58.1		61.8	CerS5
CerS6	35.5	60.4	57.4	59.0	78.1		CerS6
Similarity	CerS1	CerS2	CerS3	CerS4	CerS5	CerS6	

Supplementary Table 3. Composition of the ER-like membrane used in MD simulations. Lipid names correspond to the CHARMM forcefield convention. Counts are given as the total number of molecules of each lipid species in the cytoplasmic and ER lumenal leaflets[1,2]. Some caution needs to be taken when considering ER lipid composition due to the high likelihood of contamination from other intracellular organelles; however the composition below is currently the best approximation of ER lipid composition.

Lipid	Cytoplasmic leaflet (number of lipids)	ER lumenal leaflet (number of lipids)
SOPA	2	2
DPPA	2	2
SLPC	34	10
SOPC	20	6
PYPC	18	5
SLPE	18	28
SOPE	10	16
POPE	9	14
SOPS	8	29
POPS	2	7
SLPS	2	6
SLPG	10	10
DGPG	5	5
DEPG	5	5
PSM	25	13
NSM	9	5
LSM	10	8
Cholesterol	20	20
TOTAL	207	189

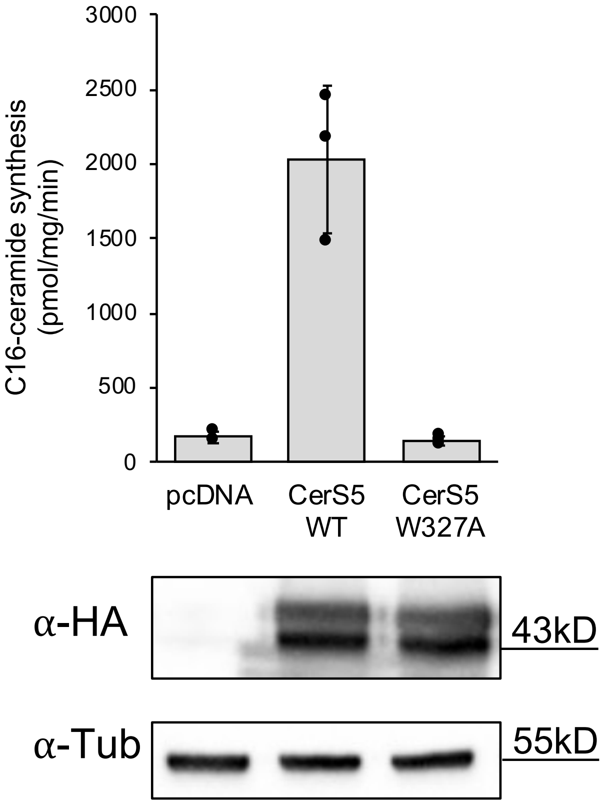

Supplementary Fig. 1. **The highly conserved residue, Trp 327 is critical for CerS activity.** Homogenates (50 µg of protein) were prepared from WT HEK cells overexpressing WT or CerS5^{W327A}. Activity was measured using 1 µg of protein and C16-CoA for 5 min. Results are means ± S.D., n = 3 for CerS5 constructs and n=2 for the empty plasmid (pcDNA). Levels of protein expression, ascertained by western blotting using an anti-HA antibody and anti-tubulin as a loading control, are shown. Molecular weight markers are indicated. Results are of a single experiment, repeated three times with similar results. Source data are provided as a Source Data file.

```
CERS2_WT      1 -------MLQTLYDYFWWERLWLPVNLTWADLE-DRDGRVYAKASDLYITLPLALLFLIVRYFFELYVATPLAALLNIKE
CERS2_D5      1 -------MLQTLYDYFWWERLWLPVNLTWADLE-DRDGRVYAKASDLYITLPLALLFLIIRFFFELFVATPLARLLNIKE
CERS2_D10     1 -------MLQTLYDYFWWERLWLPVNLTWADLE-DRDGRVYAKASDLYITLPLALLFLIIRFFFELFVATPLARLLNIKE
CERS2_D13     1 -------MLQTLYDYFWWERLWLPVNLTWADLE-DRDGRVYAKASDLYITLPLALLFLIIRFFFELFVATPLARLLNIKE
CERS2_D18     1 -------MLQTLYDYFWWERLWLPVNLTWADLE-DRDGRVYAKASDLYLTLPLALLFLIIRFLFERFIATPLARLLNIKE
CERS5_WT      1 MATAAQGPLSLLWGWLWSERFWLPENVSWADLEGPADGYGYPRGRHILSVFPLAAGIFFVRLLFERFIAKPCALCIGIED
CERS5_D5      1 MATAAQGPLSLLWGWLWSERFWLPENVSWADLEGPADGYGYPRGRHILSVFPLALGIFFLRLLFERFIAKPCALCIGIED
CERS5_D13     1 MATAAQGPLSLLWGWLWSERFWLPENVSWADLEGPADGYGYPRGRHILSVFPLALGIFFLRLLFERFIAKPCALLIGIED
CERS5_D18     1 MATAAQGPLSLLWGWLWSERFWLPENVSWADLEGPADGYGYPRGRHILSVFPLALGIFFLRLLFERFIAKPLALRLGIED
                        *. *:.::* **:*** *::*****   **  *.:. .:  .:***  ::::* :** ::*.* *  :.*::

CERS2_WT     73 KTRLRAPPNATLEHFYLTSGKQPKQVEVELLSRQSGLSGRQVERWFRRRRNQDRPSLLKKFREASWRFTFYLIAFIAGMA
CERS2_D5     73 KTRLRAPPNATLEHFYLTSGKQPKQVEVELLSRQSGLSGRQVERWFRRRRNQDRPSLLKKFREASWRFTFYLIAFIAGLA
CERS2_D10    73 KTRLRAPPNPTLEHFYLTSGKQPKQSEVELLSKQSGLSGRQVERWFRRRRNQDRPSLLKKFREASWRFTFYLIAFIAGLA
CERS2_D13    73 KTRLRAPPNPTLEHFYLTSGKQPKQSEVELLSKQSGLSGRQVERWFRRRRNQDRPSLLKKFREASWRFFFYLIAFIAGLA
CERS2_D18    73 KTRLRAPPNPTLEKFYLTSGKQPKQSEVELLAKQSGLSGRQVERWFRRRRNQDRPSLLKKFREACWRFFFYLLAFIAGLL
CERS5_WT     81 SGPYQAQPNAILEKVFISITKYPDKKRLEGLSKQLDWNVRKIQCWFRHRRNQDKPPTLTKFCESMWRFTFYLCIFCYGIR
CERS5_D5     81 SGPYQAQPNAILEKVFISITKYPDKKRLEGLSKQLDWNVRKIQCWFRHRRNQDKPPTLTKFCESMWRFTFYLCIFCYGIR
CERS5_D13    81 SGPYQAQPNAILEKVFISITKYPDKKRLEGLSKQLDWDVRKIQCWFRHRRNQDKPPTLTKFCESMWRFTFYLCIFCYGIR
CERS5_D18    81 SGPYQAQPNPILEKVFITITKYPDQKRLEGLAKQLDWDVRKIQCWFRHRRNQDKPPTLTKFCESMWRFTFYLFIFIFGLR
                .   :* **. **:. :::  * *.: .:* *::* . . *::: ***:*****:*. *.** *: *** ***  *  *:

CERS2_WT    153 VIVDKPWFYDMKKVWEGYPIQSTIPSQYWYYMIELSFYWSLLFSIASDVKRKDFKEQIIHHVATIILISFSWFANYIRAG
CERS2_D5    153 VIVDKPWFYDMKKVWEGYPIQSTIPSQYWYYMIELSFYWSLLFSIASDVKRKDFKEQIIHHLATIILISFSWFANYIRAG
CERS2_D10   153 VIVDKPWFYDMKKVWEGYPIQSTIPSQYWYYMIELSFYWSLLFSIASDVKRKDFKEQIIHHLATIILISFSWFANYIRVG
CERS2_D13   153 VIVDKPWFYDMKKVWEGYPIQSTIPSQYWYYMIELAFYWSLLFSIASDVKRKDFKEQIIHHLATIILISFSWFANYIRVG
CERS2_D18   153 VIIDKPWFWDMRKVWEGYPIQSTIPSQYWYYMLELAFYWSLLFSIASDVKRKDFKEQIIHHLATIILISFSWFANYIRIG
CERS5_WT    161 FLWSSPWFWDIRQCWHNYPFQPLSSGLYHYYIMELAFYWSLMFSQFTDIKRKDFLIMFVHHLVTIGLISFSYINNMVRVG
CERS5_D5    161 FLWSSPWFWDIRQCWHNYPFQPLSSGLYWYYIMELAFYWSLMFSQFTDIKRKDFLIMFVHHLVTIGLISFSYINNMVRVG
CERS5_D13   161 FLWSSPWFWDIRQCWHNYPFQPLSSGLYWYYIMELAFYWSLMFSQFTDIKRKDFLIMFVHHLVTIGLISFSYINNMVRVG
CERS5_D18   161 FLWLSPWFWDIRQCWYNYPFQPLTSGLYWYYILELAFYWALLFSQFTDIKRKDFLIMFVHHLVTIGLICFSYINNMVRLG
                .:  .***:*::: * .**:*.  .. * **::**:***:*:**  :*:*****   ::**:.** **.**:: * :* *

CERS2_WT    233 TLIMALHDSSDYLLESAKMFNYAGWKNTCNNIFIVFAIVFIITRLVILPFWILHCTLVYPLELYPAFFGYYFFNSMMGVL
CERS2_D5    233 TLIMALHDSSDYLLESAKMFNYAGWKNTCNNIFIVFAIVFIITRLVILPFWILYCTLVYPLELYPAFFGYYFFNSMMGVL
CERS2_D10   233 TLIMALHDSSDYLLEAAKMFNYAGWKNTCNNIFIVFAIVFIITRLVILPFWILYCTLVYPLELYPAFFGYYFFNSMMGLL
CERS2_D13   233 TLIMALHDSSDYLLEAAKMFNYAGWKNTCNNIFIVFAIVFIITRLVILPFWILYCTLVYPLELYPAFFGYYFFNLMMGLL
CERS2_D18   233 TLIMALHDAADYLLELAKMFNYAGWKNTCNNIFIVFAIVFIITRLIILPLWILYCTLVYPLELYPPFFGYWFFNLLMGIL
CERS5_WT    241 TLIMCLHDVSDFLLEAAKLANYAKYQRLCDTLFVIFSAVFMVTRLGIYPFWILNTTLFESWEIIGPYASWWLLNGLLLTL
CERS5_D5    241 TLIMCLHDVSDFLLEAAKLANYAKYQRLCDFLFVIFSIVFFITRLGIYPFWILNTTLFESWEIIGPYASWWLLNGLLLIL
CERS5_D13   241 TLIMCLHDVSDFLLEAAKLANYAKYQRLCDLLFVIFAIVFFITRLGIYPLWILWTTLFESWEIIGPYASWWLLNGLLLLL
CERS5_D18   241 TLVMLLHDVADFLLEAAKLANYAKWQRLCDLLFVIFAIVFFITRLGIYPLWILYTTLIESWEIIGPYASWWLLNGLLLIL
                **:* *** :*:*** **: *** ::. *: :*::*: **::*** * *:***  **. . *:  .: .::::* ::  *

CERS2_WT    313 QLLHIFWAYLILRMAHK-FITGKLVEDERSDREETESSEGEEAAAGGGAKSRPLANGHPILN----NNHRKND
CERS2_D5    313 QLLHIFWAYLILRMAHK-FITGKLVEDERSDREETESSEGEEAAAGGGAKSRPLANGHPILN----NNHRKND
CERS2_D10   313 QLLHIFWAFLILRMAHK-FITGKLVEDERSDREETESSEGEEAAAGGGAKSRPLANGHPILN----NNHRKND
CERS2_D13   313 QLLHIFWAFLILRMAHK-FITGKLVEDERSDREETESSEGEEAAAGGGAKSRPLANGHPILN----NNHRKND
CERS2_D18   313 QLLHIFWAFLILRMAHK-FITGKLVEDERSDREETESSEGEEAAAGGGAKSRPLANGHPILN----NNHRKND
CERS5_WT    321 QLLHVIWSYLIARIALKALIRGKVSKDDRSDVESSSEEEDVTTCTKSPCDS-SSSNGANRVNGHMGGSYWAEE
CERS5_D5    321 QLLHIIWSYLIIRIALKALIRGKVSKDDRSDVESSSEEEDVTTCTKSPCDS-SSSNGANRVNGHMGGSYWAEE
CERS5_D13   321 QLLHIFWAFLIIRIALKALIRGKVSKDDRSDVESSSEEEDVTTCTKSPCDS-SSSNGANRVNGHMGGSYWAEE
CERS5_D18   321 QLLHIFWAFLIIRIALKALIRGKVSKDDRSDVESSSEEEDVTTCTKSPCDS-SSSNGANRVNGHMGGSYWAEE
                ****::*::** *:* * :* **: **: :*:*** *.:...*.  :.: . ..* . :**   :*    ..:  ::
```

Supplementary Fig 2. Sequence alignment of WT and mPROSS designs. Amino acid multiple sequence alignment of mPROSS designed variants of CerS2 and CerS5 together with that of WT controls (CLUSTALW multiple alignments). The consensus line below the aligned sequences indicates positions of conserved amino acid where the asterisks (*) indicate identical residues, colons (:), and periods (.) indicate similar residues, with colons indicating higher similarity than periods. Mutations introduced by mPROSS are highlighted in *yellow*. Altogether 18 designs were obtained by mPROSS for each CerS, of which 4 were generated, expressed and tested for activity for CerS2 and 3 for CerS5.

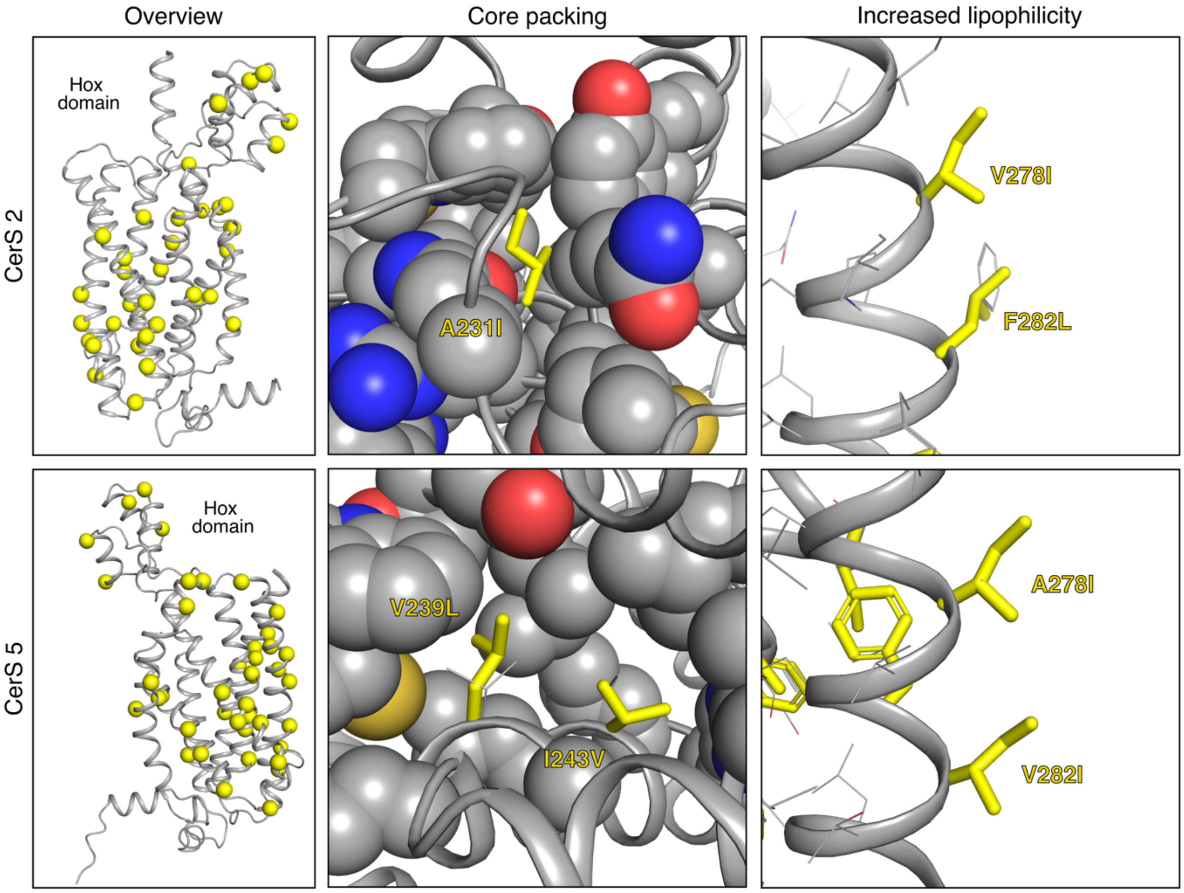

Supplementary Fig 3. CerS stabilization by mPROSS. Yellow spheres denote mutated positions. 37 and 41 substitutions were introduced in d18 of CerS2 and CerS5, respectively. Some mutations improve interhelical core packing and remove cavities (center panels). Many mutations increase lipophilicity (*right-hand* panels). Mutations are shown in yellow, wild type residues in grey.

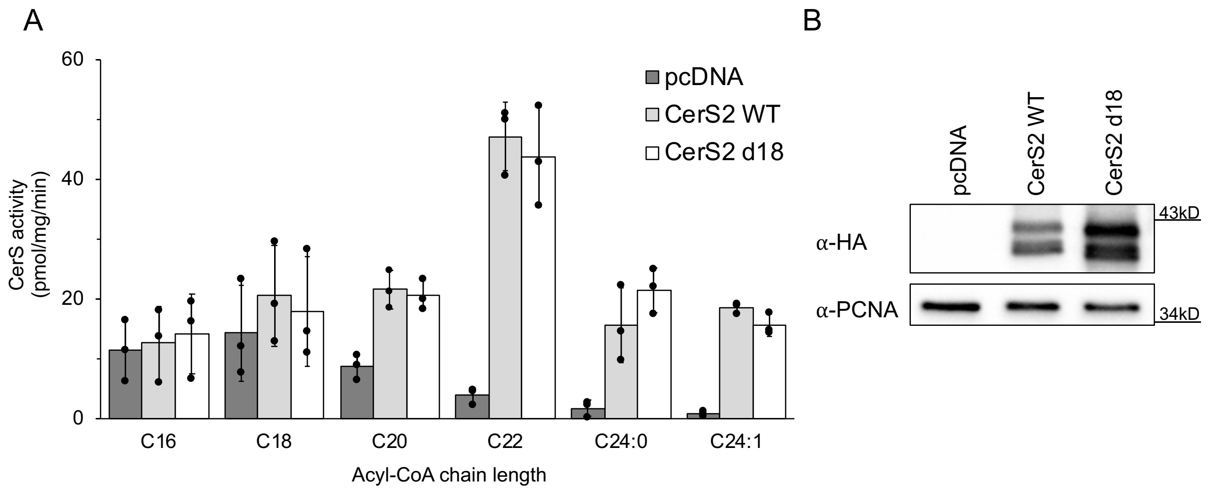

Supplementary Fig. 4. **Acyl-CoA specificity of CerS2 d18 is similar to the WT.** (A) Homogenates (40 µg of protein) from $HEK^{CerS2-/-}$ cells overexpressing an empty vector, WT or d18 CerS2, were assayed using the indicated acyl-CoA for 25 minutes. Results are means ± S.D., n = 3. (B) CerS2 expression ascertained by Western blotting using an anti-HA antibody. An anti-PCNA antibody was used as loading control. Molecular weight markers are indicated. Results are of a single experiment, repeated three times with similar results. Source data are provided as a Source Data file.

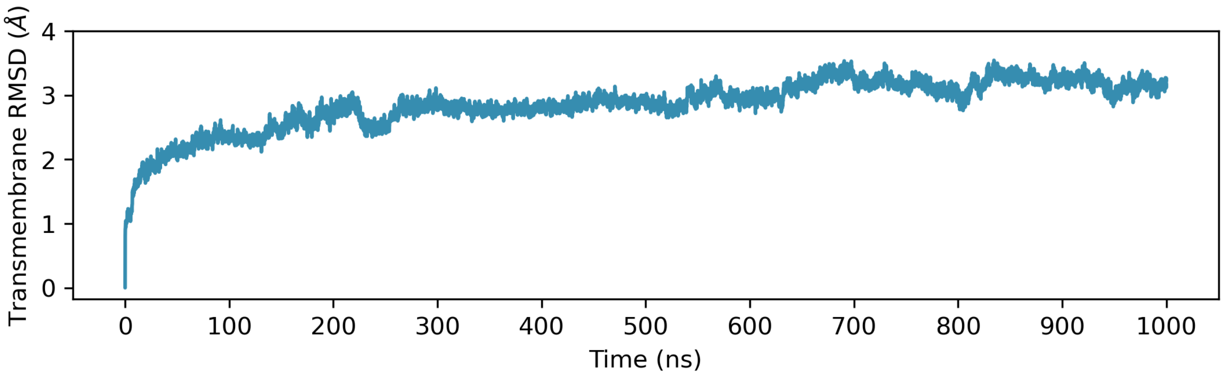

Supplementary Fig. 5. RMSD (Å) of the CerS2 TMD during production-stage MD simulations. RMSD was calculated with translation and rotation to compare the protein structure at each frame with the reference protein structure at the beginning of simulation. Source data are provided as a Source Data file.

Supplementary References

1. Andreyev, A. Y. *et al.* Subcellular organelle lipidomics in TLR-4-activated macrophages. *J Lipid Res* 51, 2785–97 (2010).

2. Bollen, I. C. & Higgins, J. A. Phospholipid asymmetry in rough- and smooth-endoplasmic-reticulum membranes of untreated and phenobarbital-treated rat liver. *Biochem J* 189, 475–480 (1980).
