# Supplementary material for: Computational design and molecular dynamics simulations suggest the mode of substrate binding in ceramide synthases
Source: Nat Commun. 2023 Apr 22;14:2330. doi: 10.1038/s41467-023-38047-x (PMC10122649; doi:10.1038/s41467-023-38047-x)
Supplement: Supplementary file 2 — Reporting Summary [file 41467_2023_38047_MOESM2_ESM.pdf]

nature portfolio

Corresponding author(s): Anthony H. Futerman

Last updated by author(s): Feb 5, 2023

Reporting Summary

Nature Portfolio wishes to improve the reproducibility of the work that we publish. This form provides structure for consistency and transparency in reporting. For further information on Nature Portfolio policies, see our Editorial Policies and the Editorial Policy Checklist.

Statistics

For all statistical analyses, confirm that the following items are present in the figure legend, table legend, main text, or Methods section.

n/a	Confirmed	
☐	☒	The exact sample size (*n*) for each experimental group/condition, given as a discrete number and unit of measurement
☒	☐	A statement on whether measurements were taken from distinct samples or whether the same sample was measured repeatedly
☒	☐	The statistical test(s) used AND whether they are one- or two-sided<br>*Only common tests should be described solely by name; describe more complex techniques in the Methods section.*
☒	☐	A description of all covariates tested
☒	☐	A description of any assumptions or corrections, such as tests of normality and adjustment for multiple comparisons
☐	☒	A full description of the statistical parameters including central tendency (e.g. means) or other basic estimates (e.g. regression coefficient) AND variation (e.g. standard deviation) or associated estimates of uncertainty (e.g. confidence intervals)
☒	☐	For null hypothesis testing, the test statistic (e.g. *F*, *t*, *r*) with confidence intervals, effect sizes, degrees of freedom and *P* value noted<br>*Give P values as exact values whenever suitable.*
☒	☐	For Bayesian analysis, information on the choice of priors and Markov chain Monte Carlo settings
☒	☐	For hierarchical and complex designs, identification of the appropriate level for tests and full reporting of outcomes
☒	☐	Estimates of effect sizes (e.g. Cohen's *d*, Pearson's *r*), indicating how they were calculated

Our web collection on statistics for biologists contains articles on many of the points above.

Software and code

Policy information about availability of computer code

Data collection	The CerS protein mPROSS designs were generated using custom software available at: https://mPROSS.weizmann.ac.il. The lipid bilayer was constructed using CHARMM-GUI (version 3.7). Molecular dynamics simulation was performed using open source software (GROMACS version 2020, source code archive DOI: 10.5281/zenodo.3562495) using the CHARMM36 forcefield.
Data analysis	Alignment of human CerS sequences was performed with Muscle 3.81.31. Similarity and identity percentages were calculated with MacVector version 18.0. Alignment of mPROSS designs and WT CerS2 and CerS5 were performed with CLUSTALW 2.1. SiteMap analysis was performed using commercial software (Schrödinger Suite version 2021-4 build 135). Molecular structure figures generated using open source softwares VMD version 1.9.3, PyMOL 2.3.5. Graphs were generated using open source software (Python 3.8.5). The Python code used to generate manuscript Figure 3B and Supplementary Figure 5 are available at https://github.com/tamir-dingjan/CerS2 with DOI: 10.5281/zenodo.7608937.

For manuscripts utilizing custom algorithms or software that are central to the research but not yet described in published literature, software must be made available to editors and reviewers. We strongly encourage code deposition in a community repository (e.g. GitHub). See the Nature Portfolio guidelines for submitting code & software for further information.

Data

Policy information about availability of data

All manuscripts must include a data availability statement. This statement should provide the following information, where applicable:

- Accession codes, unique identifiers, or web links for publicly available datasets
- A description of any restrictions on data availability
- For clinical datasets or third party data, please ensure that the statement adheres to our policy

Human CerS three-dimensional models are available at https://alphafold.ebi.ac.uk/ under the following UniProt accession codes: CerS1, P27544; CerS2, Q96G23; CerS3, Q8IU89; CerS4, Q9HA82; CerS5, Q8N5B7; CerS6, Q6ZMG9. The accession codes for the PDB structures of the hox-like domains are CerS5, PDB: 2CQX [http://doi.org/10.2210/pdb2CQX/pdb] and CerS6, PDB: 1X2M; [http://doi.org/10.2210/pdb1X2M/pdb]. The molecular dynamics simulation trajectory files and SiteMap analysis files are publicly available at https://github.com/tamir-dingjan/CerS2, DOI:10.5281/zenodo.7608937. The source data underlying Figures 2, and Supplementary Figures 1 and 4 are provided as a Source Data file.

Human research participants

Policy information about studies involving human research participants and Sex and Gender in Research.

Reporting on sex and gender	N/A
Population characteristics	N/A
Recruitment	N/A
Ethics oversight	N/A

Note that full information on the approval of the study protocol must also be provided in the manuscript.

Field-specific reporting

Please select the one below that is the best fit for your research. If you are not sure, read the appropriate sections before making your selection.

[x] Life sciences   [ ] Behavioural & social sciences   [ ] Ecological, evolutionary & environmental sciences

For a reference copy of the document with all sections, see nature.com/documents/nr-reporting-summary-flat.pdf

Life sciences study design

All studies must disclose on these points even when the disclosure is negative.

Sample size	A minimum sample size of 3 samples per experimental set is commonly used in CerS in-vitro activity (DOI: 10.1074/jbc.M307104200, DOI: 10.1074/jbc.M109.077610, DOI: 10.1074/jbc.RA118.001936).
Data exclusions	No data was excluded
Replication	All activity measurements were repeated on 3 distinct cell samples (different cell passage, different transfection day), in technical duplicates to ensure reproducibility.
Randomization	Cell culture plates for transfection were allocated randomly.
Blinding	Blinding was not relevant for this study since there is no placebo effect on cell lines.

Reporting for specific materials, systems and methods

We require information from authors about some types of materials, experimental systems and methods used in many studies. Here, indicate whether each material, system or method listed is relevant to your study. If you are not sure if a list item applies to your research, read the appropriate section before selecting a response.

Materials & experimental systems

n/a	Involved in the study
	[x] Antibodies
	[x] Eukaryotic cell lines
[x]	Palaeontology and archaeology
[x]	Animals and other organisms
[x]	Clinical data
[x]	Dual use research of concern

Methods

n/a	Involved in the study
[x]	ChIP-seq
[x]	Flow cytometry
[x]	MRI-based neuroimaging

Antibodies

Antibodies used	For western blot, we have used rabbit anti-HA (1:10,000; sigma H6908) to identify the over expressed tagged proteins, and both mouse anti-alpha tubulin (1:10,000; sigma T9026) and mouse anti-PCNA (1:500; Santa Cruz SC-56) for loading control. Secondary antibodies for detection: Goat Anti-Mouse Hrp (1:5,000; Jackson immunoResearch 115-035-003) and Goat Rabbit Hrp (1:5,000; Jackson immunoResearch 111-035-003).
Validation	Anti-HA polyclonal anti HA-tagged fusion protein: provider statement including relevant citations is available here: https://www.sigmaaldrich.com/IL/en/product/sigma/h6908<br>Anti-PCNA monoclonal anti mouse, rat, human, insect and S. pombe: provider statement including relevant citations is available here: https://www.scbt.com/p/pcna-antibody-pc10<br>Anti-alpha tubulin monoclonal anti yeast, mouse, amphibian, human, rat, chicken, fungi, bovine: provider statement including relevant citations is available here: https://www.sigmaaldrich.com/IL/en/product/sigma/t9026

Eukaryotic cell lines

Policy information about cell lines and Sex and Gender in Research

Cell line source(s)	WT cells used were commercially available Human Embryonic Kidney cells 293T (ATCC, CRL-3216).<br>CerS2 KO cells were generated in our lab and described in Tidhar et al, 2018 (DOI:https://doi.org/10.1074/jbc.RA118.001936)
Authentication	WT cell lines were not authenticated. CerS2 KO cells were authenticated by CerS2 in-vitro activity (compared with WT cells).
Mycoplasma contamination	Cells were tested when a contamination was suspected, and tested negative in all tests.
Commonly misidentified lines (See ICLAC register)	None
